# Effects of ATP and Taxifolin on Atezolizumab-Induced Renal Injury: A Biochemical, Histopathological, and Immunofluorescence Evaluation

**DOI:** 10.3390/life16071118

**Published:** 2026-07-05

**Authors:** Adil Furkan Kilic, Esra Tuba Sezgin, Gulbaniz Huseynova, Cengiz Sarigul, Mustafa Ozkaraca, Ali Gungor, Renad Mammadov, Halis Suleyman, Orhan Cimen

**Affiliations:** 1 Department of Internal Medicine, Faculty of Medicine, Atatürk University, 25240 Erzurum, Turkey; afurkan.kilic@atauni.edu.tr; 2 Anesthesia Program, Vocational School of Health Services, Erzincan Binali Yıldırım University, 24002 Erzincan, Turkey; esra.demir@erzincan.edu.tr; 3 Department of Pharmacology, Azerbaijan Medical University, AZ1022 Baku, Azerbaijan; huseynovagulbaniz04@gmail.com; 4 Department of Medical Biochemistry, Faculty of Medicine, Erzincan Binali Yıldırım University, 24100 Erzincan, Turkey; cengiz.sarigul@erzincan.edu.tr; 5 Department of Pathology, Faculty of Veterinary Medicine, Sivas Cumhuriyet University, 58140 Sivas, Turkey; mustafaozkaraca@cumhuriyet.edu.tr; 6 Laboratory and Veterinary Health Program, Vocational School of Health Services, Osmaniye Korkut Ata University, 80000 Osmaniye, Turkey; aligungor@osmaniye.edu.tr; 7 Department of Pharmacology, Faculty of Medicine, Erzincan Binali Yıldırım University, 24100 Erzincan, Turkey; rmammadov@erzincan.edu.tr; 8 Department of General Surgery, Faculty of Medicine, Erzincan Binali Yıldırım University, 24100 Erzincan, Turkey

**Keywords:** atezolizumab, nephrotoxicity, oxidative stress, ATP, taxifolin, apoptosis, antioxidant defense

## Abstract

Background: Immune checkpoint inhibitors (ICIs), particularly programmed death-ligand 1 (PD-L1) inhibitors such as atezolizumab, have significantly improved outcomes in cancer therapy. However, these agents may cause immune-related adverse effects, including nephrotoxicity associated with oxidative stress and cellular stress responses. This study aimed to investigate and comparatively evaluate the protective effects of adenosine triphosphate (ATP) and taxifolin against atezolizumab-induced renal tissue injury in rats. Methods: Animals were divided into four groups: healthy (HG), atezolizumab (ATZ), ATP + atezolizumab (ATAZ), and taxifolin + atezolizumab (TXAZ). ATP (4 mg/kg, i.p.) and taxifolin (50 mg/kg, oral) were administered for six days, while atezolizumab (10 mg/kg, i.p.) was given on days 1 and 4. On day 7, renal tissues were collected for biochemical, histopathological, and double immunofluorescence analyses. Results: Atezolizumab significantly increased malondialdehyde (MDA) levels and decreased total glutathione (tGSH), superoxide dismutase (SOD), and catalase (CAT) levels, indicating enhanced oxidative stress and impaired antioxidant defense. These changes were accompanied by tubular degeneration and increased expression of apoptotic markers. Both ATP and taxifolin significantly ameliorated these alterations; however, ATP demonstrated a more pronounced protective effect. Conclusions: In conclusion, ATP and taxifolin attenuated the biochemical, histopathological, and immunofluorescence alterations associated with atezolizumab administration. ATP exhibited a more pronounced protective effect than taxifolin under the conditions of this experimental model. Nevertheless, further experimental studies are required to elucidate the mechanisms underlying these effects.

## 1. Introduction

In recent years, immunotherapy has enabled significant advances in cancer treatment. Immune checkpoint blockade has become an important therapeutic strategy for the management of advanced malignancies. In particular, monoclonal antibodies directed against immune checkpoint molecules such as cytotoxic T-lymphocyte antigen-4 (CTLA-4) and programmed cell death protein-1 (PD-1) have demonstrated substantial clinical efficacy across a wide range of cancers [1,2]. Atezolizumab, a humanized IgG1 monoclonal antibody targeting programmed death-ligand 1 (PD-L1), has received approval from the U.S. Food and Drug Administration (FDA). It is currently administered either as a single agent or in combination with chemotherapy for the treatment of several malignancies, including urothelial carcinoma, non-small-cell lung cancer, triple-negative breast cancer, and hepatocellular carcinoma [3]. ICIs, particularly PD-1 and PD-L1 inhibitors, are associated with immune-related adverse effects that can affect multiple organs, including the skin, endocrine system, gastrointestinal tract, lungs, liver, and nervous system [4]. Although rare, kidney injury is also among the clinically significant adverse effects [5]. Renal toxicity associated with atezolizumab use may lead to immune-mediated nephritis types such as tubulointerstitial nephritis, IgA glomerulonephritis, and membranoproliferative glomerulonephritis [6]. Nephrotoxicity induced by antineoplastic drugs can cause oxidative stress through increased production of reactive oxygen species (ROS). If these ROS are not adequately cleared, this may result in increased lipid peroxidation (LPO) and impairment of the antioxidant defense system [7]. Although the exact etiology is not fully understood, ICI-related nephritis is thought to involve autoreactive T cells, expression of checkpoint receptors in non-tumor tissues, drug-specific T-cell activation, as well as cytokines and inflammation [8]. In addition, the literature reports that atezolizumab monotherapy increases intracellular calcium (Ca^2+^) and ROS levels, while decreasing ATP levels [9].

The present study examined the protective potential of ATP against atezolizumab-induced kidney injury. As the primary intracellular energy carrier, ATP is structurally composed of adenine, ribose, and three phosphate groups. ATP contributes to the control of oxidative stress by providing the energy required for maintaining antioxidant defense systems and indirectly modulates inflammatory processes [10,11]. In the literature, it has been reported that a decrease in ATP levels is a consequence of mitochondrial damage and represents one of the main mechanisms underlying the development of renal injury by leading to cell death [12].

Taxifolin, whose protective role against potential nephrotoxicity we aimed to investigate, is a potent flavonoid that reduces oxidative stress-induced cellular damage by supporting antioxidant enzyme systems and suppressing the inflammatory response [13]. It has become an effective compound in the treatment of various diseases due to its antimicrobial, anticancer, hepatoprotective, antiangiogenic, cardiovascular, and pulmonary effects [14]. Taxifolin regulates cellular energy metabolism by enhancing fatty acid β-oxidation and mitochondrial functions, thereby restoring ATP production [15]. Furthermore, it has been reported in the literature to exhibit significant nephroprotective properties against kidney injury in different experimental models by suppressing oxidative stress, inflammation, and apoptosis [16,17,18].

These findings suggest that ATP and taxifolin may have the potential to attenuate atezolizumab-induced nephrotoxicity. Moreover, to the best of our knowledge, no studies addressing this issue have been reported in the literature. Therefore, the aim of our study was to investigate and comparatively evaluate the protective effects of ATP and taxifolin against possible atezolizumab-induced renal toxicity in rats.

## 2. Materials and Methods

### 2.1. Animals

A total of 24 male Wistar albino rats, weighing 280–293 g, were included in this experimental study. The animals were supplied by the Experimental Animals Application and Research Center of Erzincan Binali Yıldırım University, Erzincan, Turkey. Before the experimental procedures, rats were acclimatized and maintained in groups of six under standardized laboratory conditions (temperature: 21–22 °C; relative humidity: 30–70%; 12 h light/12 h dark cycle), with unrestricted access to standard pellet chow and drinking water. The experimental protocol received approval from the Animal Experiments Local Ethics Committee of Erzincan Binali Yıldırım University (Meeting No. 2026/05, dated 21 May 2026). All procedures were performed in compliance with the European Parliament Directive 2010/63/EU and were consistent with the ARRIVE guidelines [19].

### 2.2. Chemicals

Chemicals used in the study were obtained as follows: atezolizumab (Tecentriq^®^, F. Hoffmann-La Roche, Basel, Switzerland; 1200 mg/20 mL) was provided by the Clinic of Medical Oncology, Mengücek Gazi Training and Research Hospital, Erzincan Binali Yıldırım University. Thiopental sodium (IE Ulagay, Istanbul, Turkey), taxifolin (Evalar, Biysk, Russia), and ATP (Zdorove Narodu, Kharkiv, Ukraine) were purchased from commercial suppliers.

### 2.3. Experimental Groups

After the acclimatization period, animals were randomly assigned to one of four groups: healthy control (HG), atezolizumab (ATZ), ATP-treated atezolizumab (ATAZ), or taxifolin-treated atezolizumab (TXAZ).

### 2.4. Experimental Procedure

Animals in the ATAZ group (*n* = 6) received ATP (4 mg/kg, i.p.) [20], while the TXAZ group (*n* = 6) was administered taxifolin (50 mg/kg, orally via gavage). The HG (*n* = 6) and ATZ (*n* = 6) groups received distilled water as a solvent. ATP was dissolved in sterile distilled water and administered immediately prior to the experiment. Atezolizumab was diluted with sterile 0.9% sodium chloride (normal saline) immediately before administration and administered intraperitoneally at a dose of 10 mg/kg. On experimental days 1 and 4, rats in the ATAZ, TXAZ, and ATZ groups received atezolizumab (10 mg/kg, i.p.) [21], which was administered 1 h after treatment with ATP, taxifolin, or the corresponding vehicle. ATP and taxifolin treatments were continued once daily for a total of six consecutive days. On day 7, all animals were euthanized by overdose anesthesia with thiopental sodium (50 mg/kg), after which kidney tissues were immediately excised for further analyses. In the collected kidney tissues, MDA and tGSH levels, as well as SOD and CAT activities, were measured. Additionally, kidney tissues were examined histopathologically and by double immunofluorescence methods. The biochemical, histopathological, and immunofluorescence findings were compared among the experimental groups.

### 2.5. Tissue MDA, tGSH, SOD, and CAT Analysis

Excised kidney samples were first rinsed with cold phosphate-buffered saline (PBS, pH 7.4) to remove residual blood and then weighed. Each tissue sample was homogenized in ice-cold PBS (1:10, *w*/*v*), followed by centrifugation at 10,000× *g* for 15 min at 4 °C. The obtained supernatants were retained for the determination of biochemical parameters. Tissue MDA, tGSH, and SOD activity were determined using commercially available assay kits (Cayman Chemical Company, Ann Arbor, MI, USA; catalog numbers 10009055, 703002, and 706002, respectively), according to the manufacturer’s instructions. CAT activity was determined using the method described by Góth [22]. Total protein concentrations were measured using the Bradford method [23]. The biochemical results were normalized to total protein content.

### 2.6. Histopathological Examinations

For histopathological examination, kidney tissues were fixed in 10% neutral-buffered formalin and processed for paraffin embedding. Serial 5-μm sections were prepared, deparaffinized, and rehydrated using graded ethanol solutions (100%, 90%, and 70%) prior to hematoxylin and eosin (H&E) staining. The sections were examined microscopically for histopathological changes. The observed alterations were evaluated semi-quantitatively as absent (0), mild (1), moderate (2), and severe (3) [24].

### 2.7. Double Immunofluorescence Method

Tissue samples embedded in paraffin were cut into 5-μm sections and mounted onto poly-L-lysine-coated slides. The sections were then deparaffinized in xylene, rehydrated using graded ethanol solutions, and washed with phosphate-buffered saline (PBS) before further processing. Antigen retrieval was performed using citrate buffer (pH 6.0) at 800 W for 2 × 5 min. After washing twice in PBS for 10 min, the sections were incubated for 10 min in PBS supplemented with 0.4% gelatin and 0.025% Triton X-100. Sections were then blocked with 5% BSA for 1 h. Following blocking, sections were incubated overnight at +4 °C with primary antibody pairs consisting of monoclonal IRE1α (Santa Cruz Biotechnology, Inc., Santa Cruz, CA, USA, sc-390960) and polyclonal Caspase-3 (Thermo Fisher, Waltham, MA, USA, PA5-114687), as well as monoclonal JNK (Santa Cruz Biotechnology, Inc., sc-7345) and polyclonal AIF (Bioss, Woburn, MA, USA, bs-0037R), at a dilution of 1:200 (All antibodies were used at a dilution of 1:200). Following PBS rinses, tissue sections were exposed for 45 min to a secondary antibody cocktail prepared in 1% BSA, consisting of goat anti-mouse FITC (ImmunoResearch, West Grove, PA, USA, 115-095-003) and anti-rabbit Alexa Fluor 595 (Cell Signaling, Danvers, MA, USA, 8889S), each used at a 1:100 dilution. The sections were then washed with PBS before being incubated for 10 min in a solution containing 10 mM CuSO_4_ and 50 mM NH_4_Cl. After rinsing with distilled water, sections were counterstained with DAPI and examined under a fluorescence microscope (Zeiss Axiolab 5 microscope, Axiocam 305 color camera, and Colibri 3 LED illumination system; Carl Zeiss, Oberkochen, German) as previously described [25]. Immunopositivity was evaluated as red fluorescence for Caspase-3 and AIF, and green fluorescence for IRE1α and JNK. Images were analyzed using Zen Blue 3.1 software, and staining intensity was scored semi-quantitatively as absent (0), mild (1), moderate (2), or severe (3) [24].

### 2.8. Statistical Analysis

Biochemical results from the experiments are expressed as mean ± SD. Differences among groups were analyzed using one-way analysis of variance (ANOVA), and when a significant effect was observed, Fisher’s LSD was applied as a post hoc test. Prior to statistical analysis, the normality of the data was examined with the Shapiro–Wilk test, and variance homogeneity was verified using Levene’s test. All analyses were carried out with SPSS for Windows (version 18.0). Differences were regarded as statistically significant when the *p* value was less than 0.05. Histopathological and double immunofluorescence data were evaluated using SPSS 20.0. For these analyses, intergroup comparisons were performed with the nonparametric Kruskal–Wallis test, and the Mann–Whitney U test was used to identify the groups responsible for observed differences (*p* < 0.05).

## 3. Results

### 3.1. Effects of ATP and Taxifolin on Renal Oxidative Status and Antioxidant Defense

**Figure 1:**
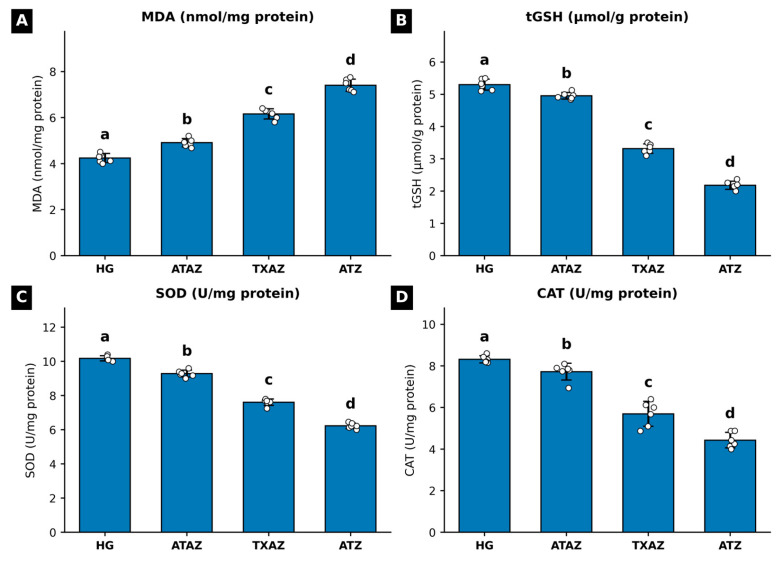
Effects of ATP and taxifolin on oxidative stress and antioxidant parameters. (**A**) MDA levels, (**B**) tGSH levels, (**C**) SOD activity, and (**D**) CAT activity in kidney tissue of experimental groups (HG, ATAZ, TXAZ, ATZ). Values are expressed as mean ± SD (*n* = 6). Statistical analysis was performed using one-way ANOVA followed by Fisher’s LSD post hoc test. Different superscript letters indicate statistically significant differences among the groups (*p* < 0.05). Abbreviations: HG, healthy group; ATAZ, ATP + atezolizumab group; TXAZ, taxifolin + atezolizumab group; ATZ, atezolizumab group.

As shown in Figure 1A, MDA levels were significantly increased in the ATZ group compared to the HG group (*p* < 0.001). In the ATAZ and TXAZ groups, MDA levels were significantly decreased compared to the ATZ group (*p* < 0.01 and *p* < 0.05, respectively). Moreover, MDA levels in the ATAZ group were significantly lower than those in the TXAZ group (*p* < 0.05).

tGSH levels were significantly decreased in the ATZ group compared to the HG group (*p* < 0.001), whereas they were significantly increased in the ATAZ and TXAZ groups compared to the ATZ group (*p* < 0.01). Additionally, tGSH levels in the ATAZ group were significantly higher than those in the TXAZ group (*p* < 0.05) (Figure 1B).

As presented in Figure 1C, SOD activity was significantly reduced in the ATZ group compared to the HG group (*p* < 0.001), while it was significantly increased in the ATAZ and TXAZ groups compared to the ATZ group (*p* < 0.01). Furthermore, SOD activity in the ATAZ group was significantly higher than that in the TXAZ group (*p* < 0.05).

Similarly, CAT activity was significantly decreased in the ATZ group compared to the HG group (*p* < 0.001), whereas it was significantly increased in the ATAZ and TXAZ groups compared to the ATZ group (*p* < 0.01). Notably, CAT activity in the ATAZ group was significantly higher than that in the TXAZ group (*p* < 0.05) (Figure 1D).

### 3.2. Histopathological Findings

**Figure 2:**
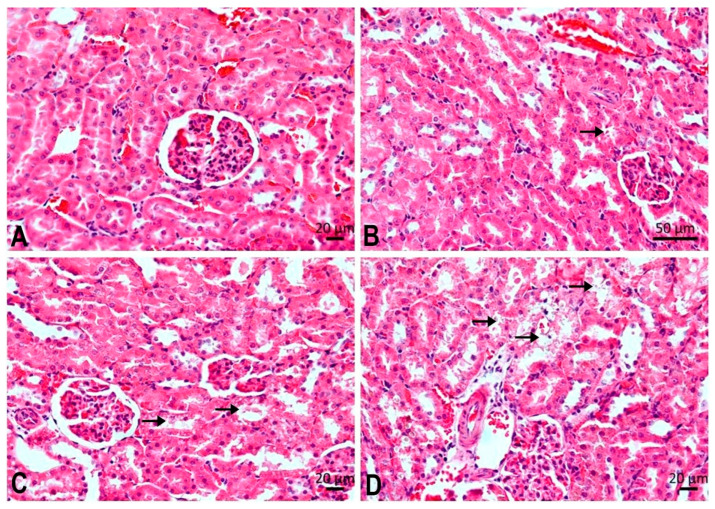
Histopathological evaluation of renal tissues. (**A**) HG group showing normal renal histology. (**B**) ATAZ group showing mild tubular degeneration. (**C**) TXAZ group showing moderate tubular degeneration. (**D**) ATZ group showing severe tubular degeneration. Arrows (→) indicate areas of tubular degeneration. H&E staining: scale bars = 20 μm (**A**,**C**,**D**) and 50 μm (**B**). Abbreviations: HG, healthy group; ATAZ, ATP + atezolizumab group; TXAZ, taxifolin + atezolizumab group; ATZ, atezolizumab group.

Histopathological examination revealed statistically significant differences among the groups (Table 1). The healthy group exhibited normal histological architecture. Varying degrees of tubular degeneration were observed in the treatment groups. This microscopic finding was mild in the ATAZ group, moderate in the TXAZ group, and severe in the ATZ group (Figure 2).

### 3.3. Double Immunofluorescence Findings

**Figure 3:**
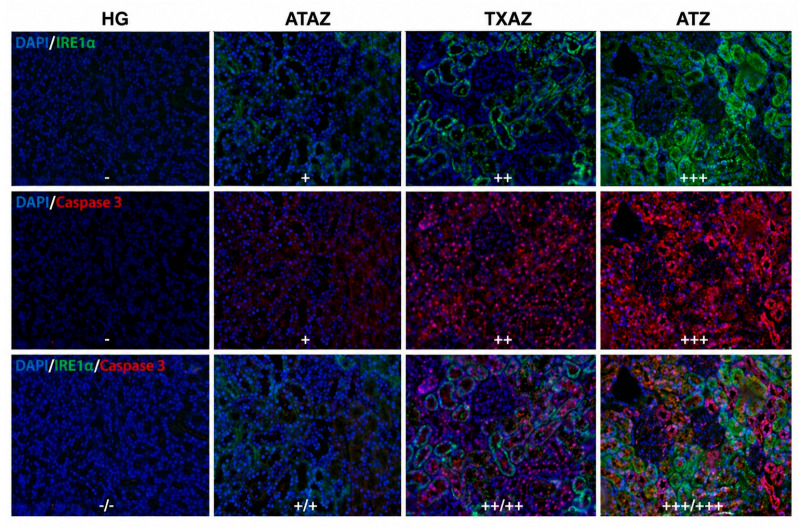
Double immunofluorescence analysis of IRE1α and Caspase-3 expression in kidney tissues. Immunoreactivity was semi-quantitatively graded as absent (−), mild (+), moderate (++), and severe (+++). Representative fluorescence micrographs showing IRE1α immunoreactivity (green), Caspase-3 immunoreactivity (red), and merged images. Nuclei were counterstained with DAPI (blue). Images are presented from left to right as HG, ATAZ, TXAZ, and ATZ groups. Minimal immunoreactivity was observed in the HG group, whereas IRE1α and Caspase-3 expression progressively increased in the ATAZ, TXAZ, and ATZ groups, with the strongest staining detected in the ATZ group. Abbreviations: HG, healthy group; ATAZ, ATP + atezolizumab group; TXAZ, taxifolin + atezolizumab group; ATZ, atezolizumab group. Immunoreactivity was evaluated semiquantitatively as −/− (negative), +/+ (mild), ++/++ (moderate), and +++/+++ (strong).

**Figure 4:**
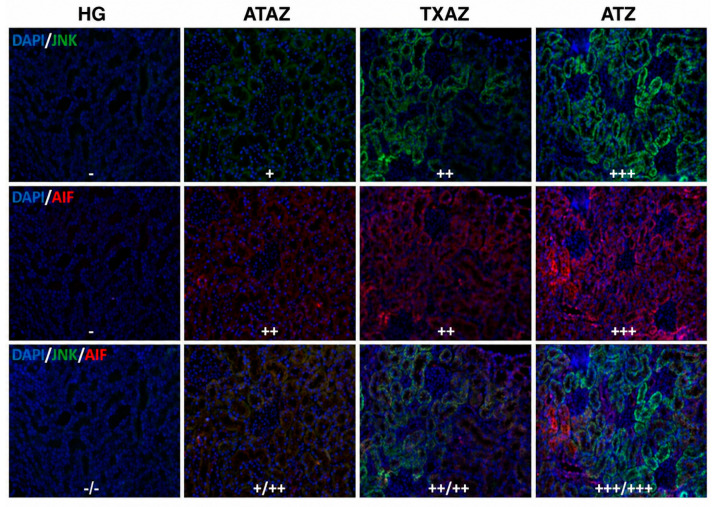
Double immunofluorescence analysis of JNK and AIF expression in kidney tissues. Immunoreactivity was semi-quantitatively graded as absent (−), mild (+), moderate (++), and severe (+++). Representative fluorescence micrographs showing JNK immunoreactivity (green), AIF immunoreactivity (red), and merged images. Nuclei were counterstained with DAPI (blue). Images are presented from left to right as HG, ATAZ, TXAZ, and ATZ groups. Minimal immunoreactivity was observed in the HG group, whereas JNK and AIF expression increased in the ATAZ, TXAZ, and ATZ groups. The highest immunoreactivity for both markers was detected in the ATZ group, while ATP and taxifolin treatment reduced staining intensity compared with the ATZ group. Abbreviations: HG, healthy group; ATAZ, ATP + atezolizumab group; TXAZ, taxifolin + atezolizumab group; ATZ, atezolizumab group. Immunoreactivity was evaluated semiquantitatively as −/− (negative), +/+ (mild), ++/++ (moderate), and +++/+++ (strong).

In kidney tissues, staining for IRE1α, Caspase-3, JNK, and AIF revealed no significant immunopositivity in the healthy group, whereas varying levels of positivity were detected in the treatment groups. IRE1α, Caspase-3, and JNK immunopositivity were mild in the ATAZ group, moderate in the TXAZ group, and severe in the ATZ group. AIF immunopositivity was moderate in the ATAZ and TXAZ groups, and severe in the ATZ group (Figure 3 and Figure 4, Table 2).

## 4. Discussion

This study investigated the potential protective effects of ATP and taxifolin against possible kidney damage induced by atezolizumab in rats, using biochemical, histopathological, and double immunofluorescence methods. The results obtained indicate that oxidative stress may occur as a result of atezolizumab administration; specifically, there was an increase in MDA levels, one of the oxidant parameters, along with significant decreases in antioxidant parameters such as tGSH, SOD, and CAT. In addition to these biochemical changes, marked histopathological damage was observed, accompanied by increased expression of stress- and apoptosis-related markers.

Neoplastic drug-induced nephrotoxicity is reported to be associated with oxidative stress resulting from increased production of ROS. Insufficient elimination of ROS may lead to increased LPO and impairment of the antioxidant defense system [7]. As is well known, MDA, the end product of LPO, is used as a biomarker for the detection of oxidative stress [26]. It has been reported in the literature that atezolizumab monotherapy increases intracellular Ca^2+^ and ROS levels, while decreasing ATP levels [9]. Liu et al. (2021) demonstrated that atezolizumab triggers mitochondria-associated cellular stress responses, which may contribute to increased oxidative stress [27]. In line with these findings, the elevated MDA levels and decreased antioxidant parameters observed in our study support the presence of oxidative stress–related renal damage. In this context, elevated MDA levels observed in the atezolizumab group indicate the development of oxidative stress. GSH, SOD, and CAT are defined as the major endogenous antioxidants that neutralize ROS [28]. Previous studies have reported that certain agents can induce kidney damage through oxidative stress, characterized by increased MDA levels along with decreased GSH levels and reduced SOD and CAT activities [29]. Consistent with these findings, the decreases in tGSH levels and SOD and CAT activities observed in the atezolizumab group in our study support the presence of impaired antioxidant defense mechanisms and oxidative stress-mediated damage. These findings indicate that oxidative stress and impairment of the antioxidant defense system may contribute to the pathogenesis of atezolizumab-associated renal tissue injury.

ATP is the primary energy source of the cell and plays a role in maintaining Ca^2+^ balance and regulating the stages of apoptosis [30]. An increase in ROS production can impair mitochondrial function, leading to alterations in Ca^2+^ homeostasis and a decrease in ATP production [31]. In this case, reduced ATP levels play a fundamental role in the development of renal damage by causing cellular dysfunction and loss of kidney function [32]. In our study, ATP administration significantly attenuated atezolizumab-induced oxidative stress and improved antioxidant parameters, including increased tGSH levels and enhanced SOD and CAT activities. ATP, although an endogenous molecule, can exert effects via purinergic receptors in the extracellular environment without being completely degraded [33]. These findings suggest that ATP may provide protection against atezolizumab-associated renal tissue injury, potentially through the attenuation of oxidative stress. Nevertheless, the underlying mechanisms were not directly elucidated in the present study. Although ATP is known to exert extracellular biological effects through purinergic (P2) receptors, the specific receptor subtypes and downstream signaling pathways involved were not investigated in the present study. Therefore, the proposed mechanism remains speculative and requires further experimental investigation. Future studies focusing on purinergic receptor signaling and related molecular pathways will be important for elucidating the nephroprotective mechanism of ATP.

Taxifolin, whose protective role against atezolizumab-induced nephrotoxicity we aimed to investigate, is a potent flavonoid that reduces oxidative stress-induced cellular damage by supporting antioxidant systems [13]. Alanazi et al. (2022) and Algefare et al. (2022) reported that elevated MDA levels in kidney injury models decreased following taxifolin administration [16,17]. Similarly, Althunibat et al. (2025) demonstrated that reduced tGSH, SOD, and CAT levels in nephrotoxicity were significantly improved with taxifolin treatment [18]. Consistent with these findings, our study also showed that oxidative stress decreased in the taxifolin group and that the redox balance improved in favor of antioxidants. However, it was observed that ATP administration was more effective than taxifolin in improving antioxidant parameters and suppressing oxidative stress.

In our study, the histopathological findings were consistent with our biochemical data. In nephrotoxicity induced by antineoplastic drugs, oxidative stress occurs as a result of increased ROS production [7]. The detection of marked tubular degeneration in the atezolizumab-only group histopathologically indicates that atezolizumab may cause structural damage in renal tissue, which is consistent with the literature [34]. In contrast, the milder damage observed in the ATAZ group was in agreement with the biochemical findings and suggests that ATP may provide protection against atezolizumab-associated renal tissue injury. The protective role of ATP in nephrotoxicity has also been reported in the literature [35]. However, the moderate level of improvement observed in the taxifolin group indicates that taxifolin partially prevents damage through its antioxidant properties, but exhibits a less pronounced protective effect compared to ATP. It has been reported in the literature that taxifolin reduces tissue damage in oxidative stress models in renal tissue [18]. Accordingly, the protective effects of taxifolin against potential damage are supported. In contrast, ATP demonstrated more pronounced protective effects than taxifolin against the renal tissue alterations observed in this experimental model.

In double immunofluorescence analyses, the absence of significant expression of IRE1α, Caspase-3, JNK, and AIF in the healthy group indicates that apoptotic and stress response pathways are not active in normal renal tissue. IRE1α is a protein that senses endoplasmic reticulum (ER) stress and regulates cellular stress, and it activates apoptotic signaling pathways under severe stress conditions [36]. Caspase-3 is a critical mediator enzyme of apoptosis [37]. JNK is an apoptosis-related molecule activated under stress conditions [38]. AIF, on the other hand, triggers caspase-independent apoptosis [39]. Compared to the HG, ATAZ, and TXAZ groups, the increased expression of these markers in the ATZ group suggests that atezolizumab administration may be associated with the activation of cellular stress responses and apoptotic processes. Especially in the ATZ group, apoptotic pathways appeared to be markedly activated. The increased AIF positivity further supports the involvement of caspase-independent apoptotic pathways in this process. The high levels of these markers in the atezolizumab-treated group are consistent with previous findings showing that oxidative stress leads to apoptosis in renal tissue [40,41,42]. In contrast, the reduced expression of these markers in the ATP-treated group suggests attenuation of apoptosis-associated pathways [35]. Taxifolin also significantly prevented the increase in these markers. This finding is consistent with literature reporting the protective effects of taxifolin in reducing cellular damage [15]. Overall, ATP demonstrated a more pronounced protective profile than taxifolin. The doses of ATP and taxifolin used in the present study were selected based on previous studies demonstrating their protective efficacy in experimental models of oxidative tissue injury. However, dose–response relationships, therapeutic windows, and pharmacokinetic characteristics were beyond the scope of the present study. Future studies investigating different dose levels and pharmacokinetic profiles are needed to optimize treatment strategies and facilitate the translation of these findings into clinical practice.

Several limitations of the present study should be acknowledged. First, the investigation was restricted to the acute phase of renal injury; therefore, the long-term effects of atezolizumab as well as the sustained protective potential of ATP and taxifolin were not evaluated. In addition, molecular analyses focused primarily on selected markers of apoptosis and cellular stress. Other signaling pathways associated with oxidative stress and inflammation, including Nrf2, NF-κB, and mechanisms related to mitochondrial biogenesis, were not examined in detail. Furthermore, renal function biomarkers, including blood urea nitrogen (BUN) and creatinine, were not assessed. Therefore, the relationship between the observed tissue alterations and functional renal impairment could not be fully established, limiting the clinical interpretation of the findings. In addition, the extracellular effects of ATP mediated via purinergic receptors and the contribution of these pathways to the observed results were not directly evaluated. Furthermore, dose–response relationships, therapeutic windows, and the pharmacokinetic characteristics of ATP and taxifolin were beyond the scope of the present study. Future studies evaluating different dose levels and pharmacokinetic profiles are warranted to optimize treatment strategies and facilitate the clinical translation of these findings. Although thiopental sodium may influence tissue energy metabolism and antioxidant status, its potential impact on intergroup comparisons was minimized by applying the same euthanasia protocol uniformly across all experimental groups. Another limitation of our study is the absence of ATP-only and taxifolin-only groups, which precluded the evaluation of the intrinsic effects of these compounds on normal renal tissue. Future studies including these groups will help to better elucidate the independent biological effects of ATP and taxifolin. It should also be acknowledged that the use of commercially available ATP and taxifolin preparations represents a limitation of the present study. Since the manufacturers did not provide detailed information regarding the chemical purity of the active compounds or Certificates of Analysis (CoA), the potential influence of product composition and excipients on the study outcomes cannot be completely excluded. Future studies using research-grade ATP and taxifolin with well-defined purity and composition would further improve the reproducibility and interpretability of the findings. Finally, the pharmacokinetic properties and administration regimen of atezolizumab under experimental conditions may not fully correspond to clinical use. Therefore, further studies with larger sample sizes, longer durations, and advanced molecular analyses are needed to validate the findings and to elucidate the underlying mechanisms in more detail.

## 5. Conclusions

This study suggests that atezolizumab may induce nephrotoxic alterations in rat kidney tissue, characterized by marked oxidative stress, suppression of the antioxidant defense system, histopathological damage, and activation of apoptotic pathways. Increased MDA levels, along with decreased tGSH, SOD, and CAT levels, indicate that the drug disrupts the redox balance in favor of oxidants. This biochemical impairment was accompanied by structural damage characterized by tubular degeneration and increased expression of IRE1α, Caspase-3, JNK, and AIF. ATP and taxifolin administration significantly improved the biochemical, histopathological, and immunofluorescence alterations induced by atezolizumab. Both agents suppressed oxidative stress, improved antioxidant parameters, reduced histopathological damage, and limited apoptotic processes. However, the protective effect of ATP was more pronounced compared to taxifolin; it exhibited a stronger effect particularly in terms of oxidative stress parameters, antioxidant status, and histopathological improvement. In conclusion, ATP and taxifolin improved the biochemical, histopathological, and immunofluorescence alterations associated with atezolizumab administration. ATP exhibited more pronounced protective effects than taxifolin in this experimental model. However, further experimental and clinical studies are needed to confirm these findings and to clarify the underlying mechanisms.

## Figures and Tables

**Table 1:** Histopathological evaluation of tubular degeneration in experimental groups.

Group	Tubular Degeneration (Score, Median [Min–Max])
HG	0 (0–0) ^a^
ATAZ	1 (0–1) ^b^
TXAZ	2 (2–3) ^c^
ATZ	3 (2–3) ^d^

Data are expressed as median (min–max). Statistical analysis was performed using the Kruskal–Wallis test to compare multiple groups, followed by the Mann–Whitney U test to identify the groups responsible for the differences. Different superscript letters indicate statistically significant differences among the groups (*p* < 0.05). Abbreviations: HG, healthy group; ATAZ, ATP + atezolizumab group; TXAZ, taxifolin + atezolizumab group; ATZ, atezolizumab group.

**Table 2:** Double immunofluorescence evaluation of IRE1α, Caspase-3, JNK, and AIF in kidney tissues.

Group	IRE1α	Caspase-3	JNK	AIF
HG	0 (0–0) ^a^	0 (0–0) ^a^	0 (0–0) ^a^	0 (0–0) ^a^
ATAZ	1 (1–2) ^b^	1 (1–2) ^b^	1 (1–2) ^b^	2 (1–2) ^b^
TXAZ	2 (1–2) ^c^	2 (2–2) ^c^	2 (1–2) ^c^	2 (1–2) ^b^
ATZ	3 (2–3) ^d^	3 (2–3) ^d^	3 (2–3) ^d^	3 (2–3) ^c^

Data are expressed as median (min–max). Statistical analysis was performed using the Kruskal–Wallis test to compare multiple groups, followed by the Mann–Whitney U test to identify the groups responsible for the differences. Different superscript letters indicate statistically significant differences among the groups (*p* < 0.05). Abbreviations: HG, healthy group; ATAZ, ATP + atezolizumab group; TXAZ, taxifolin + atezolizumab group; ATZ, atezolizumab group.

## Data Availability

The datasets supporting the conclusions of this study are contained within the article. Any additional information related to the study is available from the corresponding author upon reasonable request.

## Abbreviations

The following abbreviations are used in this manuscript:

ICIs	Immune checkpoint inhibitors
PD-L1	Programmed death-ligand 1
ATP	Adenosine triphosphate
MDA	Malondialdehyde
tGSH	Total glutathione
SOD	Superoxide dismutase
CAT	Catalase
CTLA-4	Cytotoxic T-lymphocyte antigen-4
FDA	Food and Drug Administration
ROS	Reactive oxygen species
LPO	Lipid peroxidation
ELISA	Enzyme-Linked Immunosorbent Assay
H &E	Hematoxylin and eosin

## References

[1] Ribas A., Wolchok J.D. (2018). Cancer immunotherapy using checkpoint blockade. Science.

[2] Chen Y.X., Wang Z.X., Jin Y., Zhao Q., Liu Z.X., Zuo Z.X., Xu R.H. (2023). An immunogenic and oncogenic feature-based classification for chemotherapy plus PD-1 blockade in advanced esophageal squamous cell carcinoma. Cancer Cell.

[3] Aleem A., Shah H. (2024). Atezolizumab. StatPearls.

[4] Yamaguchi T., Shimizu J., Shigematsu F., Watanabe N., Hasegawa T., Horio Y., Inaba Y., Fujiwara Y. (2024). Atezolizumab and nintedanib in patients with non-small cell lung cancer and interstitial lung disease. J. Thorac. Dis..

[5] Tascón J., Casanova A.G., Vicente-Vicente L., López-Hernández F.J., Morales A.I. (2025). Nephrotoxicity of immune checkpoint inhibitors in single and combination therapy—A systematic and critical review. Biomedicines.

[6] Frey C., Etminan M. (2024). Immune-related adverse events associated with atezolizumab: Insights from real-world pharmacovigilance data. Antibodies.

[7] Ranasinghe R., Mathai M., Zulli A. (2023). Cytoprotective remedies for ameliorating nephrotoxicity induced by renal oxidative stress. Life Sci..

[8] Moturi K., Sharma H., Hashemi-Sadraei N. (2024). Nephrotoxicity in the age of immune checkpoint inhibitors: Mechanisms, diagnosis, and management. Int. J. Mol. Sci..

[9] Quagliariello V., Passariello M., Bisceglia I., Paccone A., Inno A., Maurea C., Rapuano Lembo R., Manna L., Iovine M., Canale M.L. (2024). Combinatorial immune checkpoint blockade increases myoal expression of NLRP-3 and secretion of H-FABP, NT-Pro-BNP, interleukin-1β and interleukin-6: Biochemical implications in cardio-immuno-oncology. Front. Cardiovasc. Med..

[10] Dunn J., Grider M.H. (2020). Physiology, adenosine triphosphate. StatPearls.

[11] Swennen E.L.R., Dagnelie P.C., Bast A. (2006). ATP inhibits hydroxyl radical formation and the inflammatory response of stimulated whole blood even under circumstances of severe oxidative stress. Free Radic. Res..

[12] Santos N.A.G., Catão C.S., Martins N.M., Curti C., Bianchi M.L.P., Santos A.C. (2007). Cisplatin-induced nephrotoxicity is associated with oxidative stress, redox state unbalance, impairment of energetic metabolism and apoptosis in rat kidney mitochondria. Arch. Toxicol..

[13] Sunil C., Xu B. (2019). An insight into the health-promoting effects of taxifolin (dihydroquercetin). Phytochemistry.

[14] Liu Y., Shi X., Tian Y., Zhai S., Liu Y., Xiong Z., Chu S. (2023). An insight into novel therapeutic potentials of taxifolin. Front. Pharmacol..

[15] Jin X., Wang L., Yuan M., Tao H., Zha H., Xu Z., Liang G., Xu X., Zhou Q. (2025). Taxifolin attenuates cisplatin-induced acute kidney injury by promoting fatty acid oxidation. J. Biochem. Mol. Toxicol..

[16] Algefare A.I. (2022). Renoprotective and oxidative stress-modulating effects of taxifolin against cadmium-induced nephrotoxicity in mice. Life.

[17] Alanezi A.A., Almuqati A.F., Alfwuaires M.A., Alasmari F., Namazi N.I., Althunibat O.Y., Mahmoud A.M. (2022). Taxifolin prevents cisplatin nephrotoxicity by modulating Nrf2/HO-1 pathway and mitigating oxidative stress and inflammation in mice. Pharmaceuticals.

[18] Althunibat O.Y., Abukhalil M.H., Khwaldeh A., Abu-Zaiton A., Al-Fawaeir S. (2025). Protective effects of taxifolin against gentamicin-induced nephrotoxicity in mice: Modulation of oxidative stress, inflammation, apoptosis, and Nrf2 signaling. Naunyn Schmiedeberg’s Arch. Pharmacol..

[19] Percie du Sert N., Hurst V., Ahluwalia A., Alam S., Avey M.T., Baker M., Browne W.J., Clark A., Cuthill I.C., Dirnagl U. (2020). The ARRIVE guidelines 2.0: Updated guidelines for reporting animal research. PLoS Biol..

[20] Erhan E., Suleyman Z., Altuner D., Demir O., Gulaboglu M., Suleyman H. (2025). Protective effect of adenosine triphosphate against hydroxychloroquine ototoxicity in rats. Sci. Rep..

[21] Rattanapisit K., Phakham T., Buranapraditkun S., Siriwattananon K., Boonkrai C., Roytrakul S., Smith D.R. (2023). In Vitro and In Vivo Studies of Plant-Produced Atezolizumab as a Potential Immunotherapeutic Antibody. Sci. Rep..

[22] Góth L. (1991). A simple method for determination of serum catalase activity. Clin. Chim. Acta.

[23] Bradford M.M. (1976). A rapid and sensitive method for the quantitation of microgram quantities of protein utilizing the principle of protein–dye binding. Anal. Biochem..

[24] Gibson-Corley K.N., Olivier A.K., Meyerholz D.K. (2013). Principles for valid histopathologic scoring in research. Vet. Pathol..

[25] Buchwalow I.B., Böcker W. (2010). Immunohistochemistry: Basics and Methods.

[26] Cordiano R., Di Gioacchino M., Mangifesta R., Panzera C., Gangemi S., Minciullo P.L. (2023). Malondialdehyde as a potential oxidative stress marker for allergy-oriented diseases: An update. Molecules.

[27] Liu Z., Wang H., Hu C., Wu C., Wang J., Hu F., Zhang W. (2021). Targeting autophagy enhances atezolizumab-induced mitochondria-related apoptosis in osteosarcoma. Cell Death Dis..

[28] Pena E., El Alam S., Siques P., Brito J. (2022). Oxidative stress and diseases associated with high-altitude exposure. Antioxidants.

[29] Sedky A., Famurewa A.C. (2024). Anti-ischemic drug trimetazidine blocks mercury nephrotoxicity by suppressing renal redox imbalance, inflammatory stress and caspase-dependent apoptosis in rats. Drug Chem. Toxicol..

[30] Espinoza N., Papadopoulos V. (2025). Role of mitochondrial dysfunction in neuropathy. Int. J. Mol. Sci..

[31] Eftekharpour E., Fernyhough P. (2022). Oxidative stress and mitochondrial dysfunction associated with peripheral neuropathy in type 1 diabetes. Antioxid. Redox Signal..

[32] Bhargava P., Schnellmann R.G. (2017). Mitochondrial energetics in the kidney. Nat. Rev. Nephrol..

[33] Patrakeeva V.P. (2025). The role of extracellular ATP in the regulation of functional cell activity. Cell Tissue Biol..

[34] Xipell M., Victoria I., Hoffmann V., Villarreal J., García-Herrera A., Reig O., Rodas L., Blasco M., Poch E., Mellado B. (2018). Acute tubulointerstitial nephritis associated with atezolizumab, an anti-programmed death-ligand 1 (PD-L1) antibody therapy. Oncoimmunology.

[35] Yeter B., Suleyman Z., Bulut S., Cicek B., Coban T.A., Demir O., Suleyman H. (2025). Effect of adenosine triphosphate on methylphenidate-induced oxidative and inflammatory kidney damage in rats. Drug Chem. Toxicol..

[36] Ron D., Walter P. (2007). Signal integration in the endoplasmic reticulum unfolded protein response. Nat. Rev. Mol. Cell Biol..

[37] Asadi M., Taghizadeh S., Kaviani E., Vakili O., Taheri-Anganeh M., Tahamtan M., Savardashtaki A. (2022). Caspase-3: Structure, function, and biotechnological aspects. Biotechnol. Appl. Biochem..

[38] Bode A.M., Dong Z. (2007). The functional contrariety of JNK. Mol. Carcinog..

[39] Susin S.A., Lorenzo H.K., Zamzami N., Marzo I., Snow B.E., Brothers G.M., Mangion J., Jacotot E., Costantini P., Loeffler M. (1999). Molecular characterization of mitochondrial apoptosis-inducing factor. Nature.

[40] Qi K., Mu Y., Hu Y., Li J., Liu J. (2025). Comprehensive landscape of cell death mechanisms: From molecular cross-talk to therapeutic innovation in oncology. Front. Cell Dev. Biol..

[41] Vavrušáková B., Krejčí L., Pečinka L., Selingerová I., Uher M., Holánek M., Svoboda M. (2026). Immunomodulatory mechanisms of endoplasmic reticulum stress in the tumor immune microenvironment and prediction of treatment response in HER2-positive breast cancer. Res. Sq..

[42] Izadpanah A., Willingham K., Chandrasekar B., Alt E.U., Izadpanah R. (2023). Unfolded protein response and angiogenesis in malignancies. Biochim. Biophys. Acta Rev. Cancer.

